# Beyond Primary Epilepsy: Fahr's Syndrome Secondary to Congenital Parathyroid Agenesis Presenting With Generalised Seizures in a Young Adult

**DOI:** 10.7759/cureus.116181

**Published:** 2026-09-13

**Authors:** Maheen Bhangwar Baloch, Asim Ahmad, Hammad Buksh Ilahi

**Affiliations:** 1 Internal Medicine, Leeds Teaching Hospitals NHS Trust, Leeds, GBR; 2 Medicine, Ziauddin University, Karachi, PAK

**Keywords:** bilateral basal ganglia calcification, congenital hypoparathyroidism, fahr's syndrome, generalised tonic clonic seizures, hypocalcaemia

## Abstract

Symmetrical, bilateral intracranial calcifications, commonly involving the basal ganglia, constitute the characteristic features of Fahr's syndrome, which is an uncommon neurometabolic condition. Its incidence in conjunction with congenital parathyroid agenesis, however, has rarely been reported. The present case emphasises how crucial it is to identify the metabolic origins of intracranial calcification and differentiate Fahr's syndrome from primary familial brain calcification to prevent repeated neurological consequences through adequate therapy.

A 26-year-old man with congenital absence of the parathyroid gland presented to the emergency department after a 10-minute generalised tonic-clonic seizure, preceded by twitching of the perioral muscles. Examination revealed continuous perioral twitching, drowsiness in the postictal state and a positive Chvostek sign. Although the serum 25-hydroxyvitamin D (25-OH vitamin D) level remained within the normal range (88 nmol/L), laboratory tests revealed severe hypocalcaemia (1.44 mmol/L), hyperphosphatemia (1.95 mmol/L) and undetectable parathyroid hormone (<0.1 pmol/L), supporting hypoparathyroidism as the underlying cause of his symptoms. Non-contrast computed tomography (CT) of the head demonstrated typical bilateral symmetric basal ganglia calcifications, consistent with Fahr's syndrome, and an electrocardiogram (ECG) revealed QT interval prolongation. Intravenous calcium, oral calcium supplements, and alfacalcidol were used to treat the patient; this led to the correction of hypocalcaemia, full resolution of neuromuscular symptoms and no more seizures while the patient was in the hospital.

This case demonstrates the rare correlation between Fahr's syndrome and congenital parathyroid agenesis that manifests in early adulthood. It highlights how crucial it is to take reversible metabolic factors into account when young adults exhibit new-onset seizures. Adequate vitamin D stores alone cannot maintain calcium homeostasis in individuals with congenital hypoparathyroidism, as evidenced by the significant hypocalcaemia that developed despite normal vitamin D status due to lacking parathyroid hormone.

## Introduction

Fahr's syndrome is an exceptionally rare neurological disease with a prevalence of about < 1/1,000,000. It is characterised by bilateral, intracranial calcifications in the basal ganglia, dentate nucleus and cerebral cortex [1]. Neurologist Carl Theodor Fahr discovered it in 1930 [2]. It is important to distinguish it from Fahr's disease, which has a distinctive pathological pattern of bilateral non-arteriosclerotic vascular calcification of the striopallidodentate system with varying ferro-calcareous concretion deposition in the cortical sulci, thalamus, cerebral white matter and cerebellum and is often hereditary with no identifiable secondary causes [3]. Our case comes from a patient with the congenital absence of parathyroid glands, which play a key role in the pathophysiology of Fahr's syndrome. Therefore, it is fundamentally different from Fahr's disease and is a very rare presentation. From extrapyramidal movement disorders and seizures to neuropsychiatric diseases, such as hallucinations and cognitive impairment, clinical manifestations are varied [1]. Chronic metabolic disorders are usually the cause of these calcifications, which frequently take 8 to 10 years of exposure to parathyroid dysfunction to show up radiographically [2]. New studies reveal a particular clinical 'constellation' in the PHP-1b subtype, where Fahr's syndrome is linked to hypokalaemia because renal potassium channels' cyclic AMP (cAMP) signalling is compromised [1]. Additionally, the disruption of the basal ganglia's function as an inhibitory circuit may cause seizures in these patients, requiring cautious pharmaceutical therapy.

## Case presentation

A 26-year-old man with a known history of congenital absence of the parathyroid glands presented to the emergency department following a generalised tonic-clonic seizure lasting approximately 10 minutes. According to witnesses, the seizure was preceded by twitching around the mouth and facial muscles. There was no history of preceding fever, head trauma, alcohol use, or recreational drug use. The patient had been receiving 25-hydroxyvitamin D (25-OH vitamin D) supplementation for congenital hypoparathyroidism.

On examination following seizure termination, the patient was drowsy but arousable with a postictal state. There was perioral muscle twitching and a positive Chvostek sign consistent with neuromuscular irritability secondary to hypocalcaemia. Cardiovascular and respiratory examinations were unremarkable.

Electrocardiogram (ECG) was suggestive of hypocalcaemia (Figure 1). Laboratory tests showed hypocalcaemia (1.44 mmol/L, reference level: 2.20-2.60 mmol/L), hyperphosphatemia (1.95 mmol/L, reference level: 0.8-1.50 mmol/L), a low parathyroid hormone (PTH) level (<0.1 pmol/L, reference level: 1.3-9.3 pmol/L), and normal vitamin D level (88 nmol/L, reference level >50 nmol/L) (Table 1). A non-contrast computed tomography (CT) scan of the head indicated bilateral symmetric calcifications in the basal ganglia (Figure 2).

**Figure 1:**
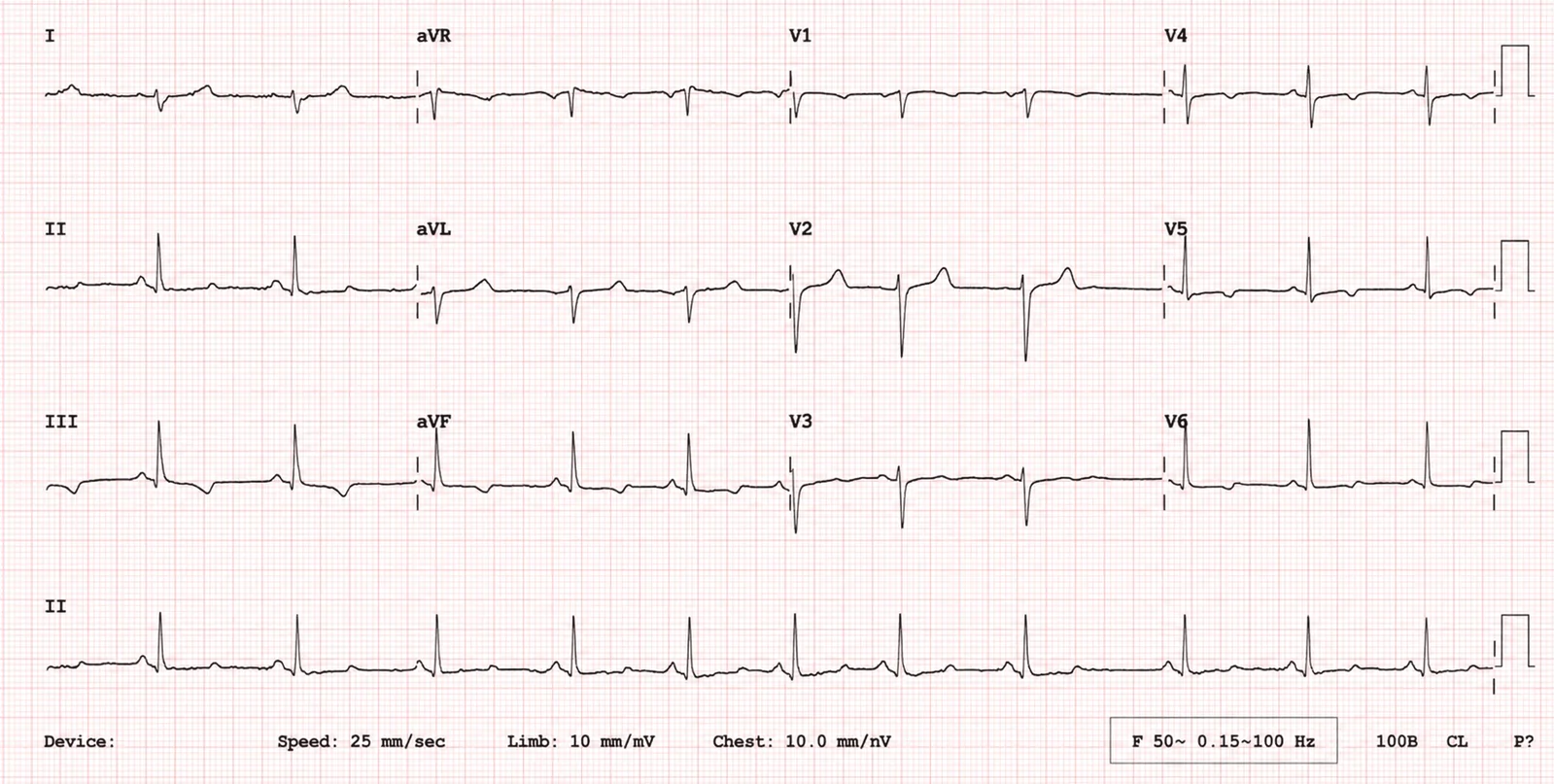
Electrocardiogram (ECG)

**Table 1:** Laboratory findings of our patient throughout hospital stay eGFR: estimated glomerular filtration; CRP: C-reactive protein; ALT: alanine aminotransferase; TSH: thyroid-stimulating hormone; 25-OH vitamin D: 25-hydroxyvitamin D; PTH: parathyroid hormone; ALP: alkaline phosphatase; T4: thyroxine

Parameter	Reference range	On discharge	Day 10	Day 9	Day 8	Day 7	Day 6	Day 5	Day 4	Day 3	Day 2	Day 1
Bicarbonate (mmol/L)	22-29	-	29	32	32	29	32	29	26	29	27	29
Albumin (g/L)	35-50	40	39	39	37	39	36	38	42	37	39	45
ALP (U/L)	30-130	60	60	63	57	61	59	69	68	63	72	74
Adjusted calcium (mmol/L)	2.20-2.60	2.09	1.91	1.77	1.75	1.69	1.68	1.7	1.59	1.5	1.5	1.44
Calcium (mmol/L)	2.2-2.6	2.08	1.88	1.74	1.69	1.66	1.6	1.66	1.61	1.44	1.47	1.51
Globulin (g/L)	-	34	35	35	31	33	34	32	31	32	31	38
Phosphate (mmol/L)	0.8-1.50	1.86	1.86	2.08	2.04	1.96	2.16	1.88	2.13	2.11	2.02	1.95
Total protein (g/L)	60-80	74	74	74	68	72	70	70	73	69	70	83
CRP (mg/L)	0-5	-		-	3	-	-	4	6	6	-	-
Chloride (mmol/L)	95-108	-		99	99	100	100	100	101	102	102	99
Free T4 (pmol/L)	7.8-21.0	-		-	-	-	-	-	-	-	-	11
ALT (U/L)	0-45	-		47	39	-	18	16	20	16	-	-
Total bilirubin (µmol/L)	<21	-		10	11	11	11	11	10	-	-	-
Magnesium (mmol/L)	0.7-1.0	-	0.67	0.66	-	0.65	0.67	0.65	0.68	0.63	-	0.73
PTH (pmol/L)	1.3-9.3	-		-	-	-	-	-	-	-	-	<0.1
TSH (mU/L)	0.35-4.7	-		-	-	-	-	-	-	-	-	1.1
25-OH vitamin D (nmol/L)	>50	-		-	-	-	-	-	-	-	-	88
Sodium (mmol/L)	133-146	-	138	139	142	142	142	142	143	141	140	140
Potassium (mmol/L)	3.5-5.3	3.7	3.6	3.7	4	4.3	4	3.5	3.4	4.2	4.4	4
Urea (mmol/L)	2.5-7.8	4	3.8	3.3	2.5	3.3	3.3	3.3	3.4	3.5	4.5	5.4
Creatinine (µmol/L)	65-114	85	84	85	89	82	94	82	78	86	79	88
eGFR (mL/min/1.73 m²)	-	>90	>90	>90	>90	>90	>90	>90	>90	>90	>90	>90

**Figure 2:**
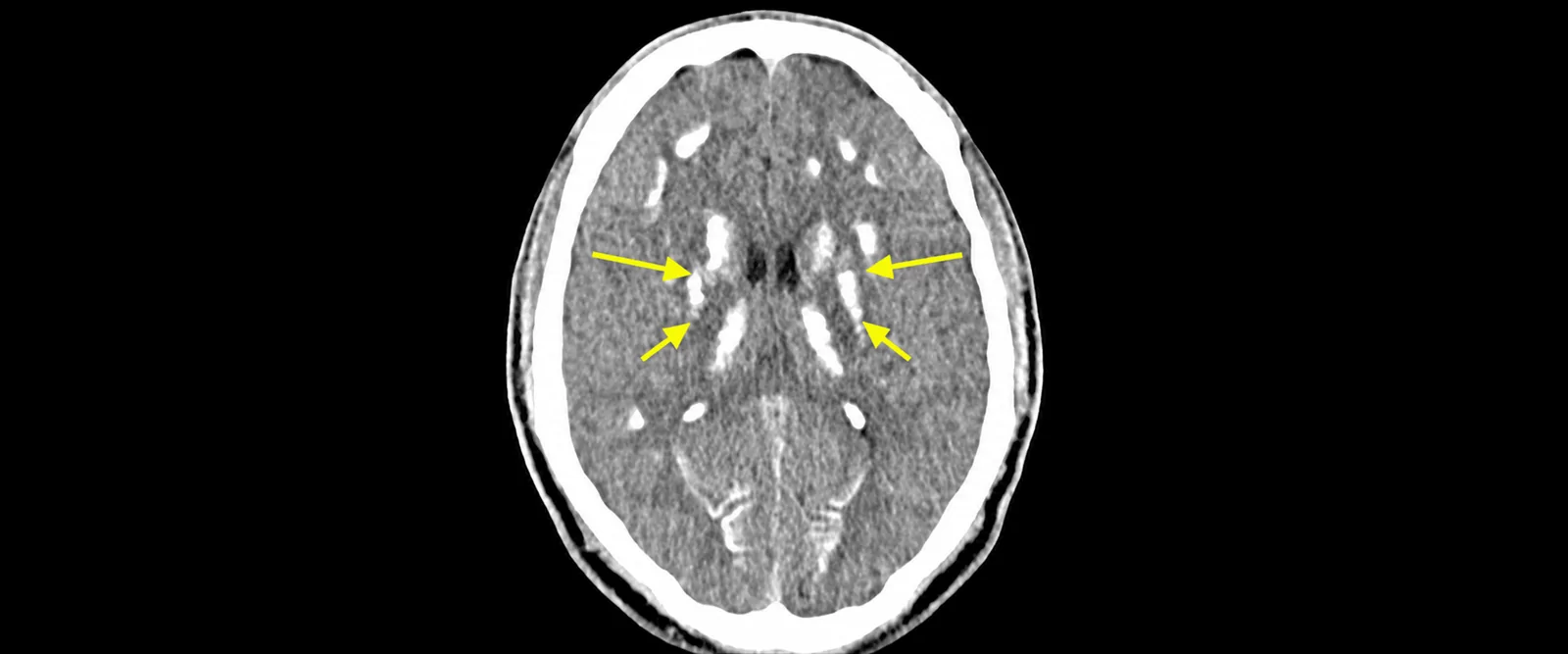
Non-contrast CT head demonstrating bilateral symmetrical basal ganglia calcifications (yellow arrows) CT: computed tomography

The patient was admitted for the management of severe symptomatic hypocalcaemia. He received intravenous calcium with continuous electrocardiographic (ECG) monitoring to detect potential cardiac arrhythmias associated with hypocalcaemia and calcium replacement.

Treatment with alfacalcidol was initiated, and the dose was gradually titrated upward according to the biochemical response. Oral calcium supplementation was also commenced. Serum corrected calcium, phosphate, and magnesium concentrations were monitored daily, and treatment was adjusted to achieve a stable serum calcium concentration while avoiding hypercalcaemia and hyperphosphatemia. Magnesium was replaced as required.

Following correction of hypocalcaemia, the patient's perioral twitching resolved completely, and no further seizures occurred during hospitalisation.

He was discharged home with oral calcium supplements and alfacalcidol in a stable condition with no recurrence of seizures. Arrangements were made for close outpatient follow-up with the endocrinology team.

## Discussion

Secondary basal ganglia calcification, another name for Fahr's syndrome, is an uncommon neurological condition. Fahr's syndrome is secondary to the identified metabolic or endocrine problems, with hypoparathyroidism being the most common cause, in contrast to primary familial brain calcification (Fahr's disease), which is caused by pathogenic mutations in genes such as *SLC20A2*, *PDGFB,* *PDGFRB*, and *XPR1 *[4,5].

It is unclear exactly what causes cerebral calcification in hypoparathyroidism. Chronic hypocalcaemia and hyperphosphatemia raise the calcium-phosphate product, which causes calcium phosphate to gradually deposit in the basal ganglia, perivascular spaces, and cerebral small arteries. According to histopathological investigations, these deposits are mostly composed of calcium phosphate, with trace amounts of magnesium and other minerals [5,6].

Seizures, movement abnormalities, parkinsonism, cerebellar dysfunction, cognitive impairment, psychiatric symptoms, and extrapyramidal characteristics are just a few of the diverse neurological manifestations. One of the most common presenting symptoms of hypoparathyroidism-associated Fahr's syndrome is seizures, which are typically brought on by acute hypocalcaemia rather than the cerebral calcifications. Thus, early hypocalcaemia correction frequently leads to total seizure resolution without the requirement for long-term antiseizure medication [7,8].

Perioral twitching is a classic sign of neuromuscular irritability brought on by hypocalcaemia, before the generalised tonic-clonic seizure that our patient had. The biochemical analysis confirmed congenital hypoparathyroidism, which demonstrated prolonged hypocalcaemia, hyperphosphatemia and undetectable serum parathyroid hormone despite sufficient vitamin D status. Extensive bilateral symmetrical calcification affecting the deep brain regions and basal ganglia was seen on CT, which is consistent with Fahr's syndrome caused by long-term hypoparathyroidism. Similar cases have been documented in the literature, where seizures were the first symptom that led to the diagnosis of basal ganglia calcification and underlying hypoparathyroidism [8-10].

Because non-contrast CT is more sensitive than magnetic resonance imaging at identifying cerebral calcification, it continues to be the preferred imaging modality. Bilateral symmetrical calcification of the globus pallidus, putamen, caudate nuclei, thalami, dentate nuclei, and subcortical white matter are typical findings. Before identifying primary familial brain calcification, a thorough metabolic assessment that includes serum calcium, phosphate, magnesium, vitamin D, and parathyroid hormone is necessary to detect treatable secondary causes [4,5].

The goal of treatment is to address the underlying metabolic problem rather than the calcifications, which are typically permanent. Intravenous calcium replacement is necessary for acute symptomatic hypocalcaemia, while oral calcium and active vitamin D analogues, such as calcitriol or alfacalcidol, are used for long-term treatment. To keep calcium within the low-normal range while reducing the risk of hypercalciuria and nephrocalcinosis, routine monitoring of serum calcium, phosphate, magnesium, renal function, and urine calcium is advised [8,11].

## Conclusions

The present case emphasises how crucial it is to identify severe hypocalcaemia as a potentially fatal consequence of congenital hypoparathyroidism. Seizures and neuromuscular irritability may be signs of poor calcium homeostasis rather than a primary neurological condition in individuals with a history of parathyroid hormone insufficiency. Clinical features such as perioral twitching, a positive Chvostek sign, a prolonged QT interval on ECG, and characteristic biochemical abnormalities should be promptly recognised and treated.

Recurrent neurological and cardiac problems can be avoided by early detection and intravenous calcium correction of hypocalcaemia, followed by suitable long-term care with active vitamin D analogues and calcium supplementation. To preserve biochemical stability and lower the likelihood of severe symptomatic episodes, this case also highlights the necessity of routine monitoring of calcium, phosphate, magnesium, and vitamin D levels in individuals with congenital hypoparathyroidism. Further evidence of the possible long-term effects of chronic calcium-phosphate imbalance and the significance of ongoing endocrinology follow-up comes from basal ganglia calcifications on neuroimaging.
